# Shear Stress and the AMP-Activated Protein Kinase Independently Protect the Vascular Endothelium from Palmitate Lipotoxicity

**DOI:** 10.3390/biomedicines12020339

**Published:** 2024-02-01

**Authors:** Asker Y. Khapchaev, Alexander V. Vorotnikov, Olga A. Antonova, Mikhail V. Samsonov, Ekaterina A. Shestakova, Igor A. Sklyanik, Alina O. Tomilova, Marina V. Shestakova, Vladimir P. Shirinsky

**Affiliations:** 1 Institute of Experimental Cardiology Named after Academician V.N. Smirnov, National Medical Research Center of Cardiology Named after Academician E.I. Chazov, Moscow 121552, Russia; loa_lu@mail.ru (O.A.A.); mvs.laba@gmail.com (M.V.S.); shirinsky@gmail.com (V.P.S.); 2 Diabetes Institute, Endocrinology Research Center, Moscow 117036, Russia; katiashestakova@mail.ru (E.A.S.); sklyanik.igor@gmail.com (I.A.S.); a.o.gavrilova@list.ru (A.O.T.); shestakova.mv@gmail.com (M.V.S.)

**Keywords:** vascular endothelium, AMP-activated protein kinase, shear stress, lipotoxicity, free fatty acid

## Abstract

Saturated free fatty acids are thought to play a critical role in metabolic disorders associated with obesity, insulin resistance, type 2 diabetes (T2D), and their vascular complications via effects on the vascular endothelium. The most abundant saturated free fatty acid, palmitate, exerts lipotoxic effects on the vascular endothelium, eventually leading to cell death. Shear stress activates the endothelial AMP-activated protein kinase (AMPK), a cellular energy sensor, and protects endothelial cells from lipotoxicity, however their relationship is uncertain. Here, we used isoform-specific shRNA-mediated silencing of AMPK to explore its involvement in the long-term protection of macrovascular human umbilical vein endothelial cells (HUVECs) against palmitate lipotoxicity and to relate it to the effects of shear stress. We demonstrated that it is the α1 catalytic subunit of AMPK that is critical for HUVEC protection under static conditions, whereas AMPK-α2 autocompensated a substantial loss of AMPK-α1, but failed to protect the cells from palmitate. Shear stress equally protected the wild type HUVECs and those lacking either α1, or α2, or both AMPK-α isoforms; however, the protective effect of AMPK reappeared after returning to static conditions. Moreover, in human adipose microvascular endothelial cells isolated from obese diabetic individuals, shear stress was a strong protector from palmitate lipotoxicity, thus highlighting the importance of circulation that is often obstructed in obesity/T2D. Altogether, these results indicate that AMPK is important for vascular endothelial cell protection against lipotoxicity in the static environment, however it may be dispensable for persistent and more effective protection exerted by shear stress.

## 1. Introduction

Increased plasma levels of saturated free fatty acids (FFAs) are typical in obesity and are thought to play a critical role in the development of metabolic disorders, type 2 diabetes (T2D), and cardiovascular complications [1,2]. Increased fat mass in obesity is associated with vascular rarefication, decreased circulation, and hypoxia [3,4] which promote chronic inflammation and insulin resistance in adipose tissue [5]. This launches a vicious circle of unregulated lipolysis and increased production of FFA by adipose tissue [6], which accumulates as ectopic fat in peripheral tissues leading to hepatic and muscle insulin resistance, reduced glucose uptake, and hyperglycemia followed by T2D [7].

The vascular endothelium is considered the primary target of elevated circulating FFAs which contribute to endothelial dysfunction and promote metabolic and cardiovascular pathologies [8,9,10,11]. Palmitic acid (PA), the most abundant saturated fatty acid in Western diets, is often used in in vitro studies to reproduce the lipotoxic effects of FFA on endothelial cells [5]. Multiple reports indicate that in vitro, moderate to high concentrations of FFA (0.5–0.7 mM and higher) induce endothelial dysfunction and compromise endothelial cell viability via oxidative stress, inflammation, and apoptosis [5,12,13,14,15,16,17,18,19,20].

In an in vivo setting, endothelial cells (EC) encounter blood flow-mediated shear stress of variable intensity and pattern, which has profound effects on multiple EC functions including gene expression and metabolism [21,22,23,24,25,26,27]. Laminar pulsatile shear stress generated in straight vascular regions is protective for the endothelium, whereas turbulent/oscillatory shear stress in branches of the conduit arteries is associated with atherogenesis [21,23,26,28,29]. In particular, laminar shear stress confers protection against FFA lipotoxicity via decreased ROS generation, downregulation of inflammatory cascades, and antiapoptotic action [27,30,31,32,33,34].

A shear stress-mediated anti-inflammatory protective role has been attributed to the AMP-activated protein kinase (AMPK) which is activated in endothelial cells subjected to flow [32,33,35]. AMPK is a critical cellular energy sensor and regulator. It is activated by an increase in cellular AMP levels and signals a falling energy state to upregulate nutrient catabolism and mitochondrial function [36,37]. AMPK activity is lower in obesity and T2D [38], consistent with high energy input and low energy expenditure due to the sedentary lifestyle of these individuals. In cardiac microvascular endothelial cells, PA has been shown to downregulate AMPK, decrease eNOS activity, increase oxidative stress, and reduce endothelial cell viability [39].

Even in the absence of mechanical stimulation, a chemically or genetically activated AMPK protects endothelial cells from oxidative stress and lipotoxicity, critically contributing to cell survival [5,12,13,38,40,41,42]. AMPK is involved in mitochondrial fission and mitophagy [43], upregulates mitochondrial uncoupling protein-2 (UCP-2) expression [13]), counteracts excessive production of reactive oxygen species (ROS) and/or nitric oxide (NO) [41], and inhibits NF-kB activation [40], all implicating AMPK in cellular defense mechanisms.

Endothelial cells express two AMPK catalytic subunits, α1 and α2. Though α1 is more abundant, the functional properties of these isoforms are only partially redundant in endothelial cells [44,45,46,47,48,49]. Currently, it is poorly understood which of the endothelial AMPK-α isoforms is responsible for resisting excessive FFA challenge. The specific roles of AMPK-α isoforms in protecting endothelial cells from FFA lipotoxicity under shear stress were neither elucidated.

Here, we report that in standard static cell culture conditions, basal AMPK-α1 activity attenuates the toxic effects of excessive palmitate on endothelial cells, whereas elevated AMPK-α2 augments palmitate toxicity. Efficient knockdown of both AMPK α1 and α2 isoforms in endothelial cells does not abrogate the protective effects of shear stress in the presence of pathologic levels of palmitate, suggesting that AMPK is dispensable for shear stress-activated mechanisms that counteract lipotoxicity. We confirm that pulsatile laminar shear stress potently protects both macrovascular and microvascular endothelial cells from the high-palmitate insult, while this protection ceases when the flow stops.

## 2. Materials and Methods

### 2.1. Materials

Lentiviral empty pSIH3–CPHB vector was obtained from Mona (Moscow, Russia). pMD2.G (a gift from Dr. Didier Trono, Addgene plasmid #12259; http://n2t.net/addgene:12259, accessed on 29 January 2024) and pCMV-dR8.91 (kindly provided by Dr. A. Shevelev, NMRCC) packaging plasmids were used for assembly of viral particles. Oligonucleotides were ordered from Syntol (Moscow, Russia). The enzymes for DNA cloning were obtained from SybEnzyme (Novosibirsk, Russia). Thermo (Waltham, MA, USA) mini-spin columns and Qiagen (Hilden, Germany) gravity flow columns were used for plasmid DNA isolation from bacterial cultures (DH5alpha *E. coli* strain). Ibidi µ-Slides I0.4 and yellow-green perfusion sets (#10964, Ibidi, Gräfelfing, Germany) were used for cell cultivation under flow conditions. Palmitic acid (PA, #9767-5G) and fatty acid-free bovine serum albumin (BSA, #A8806) were from Sigma-Aldrich (St. Louis, MO, USA) and Biosera (#PM-T1727, Cholet, France). Biotinylated 10F3B2 monoclonal antibody against cell adhesion molecule ICAM-1, Annexin-V:FITC assay kit (Bio-Rad, Hercules, CA, USA, #ANNEX100F), and Active Caspase-3 FITC Mab Apoptosis Kit I (BD Pharmingen, Franklin Lakes, NJ, USA, #550480) were used for flow cytofluorimetry.

Dulbecco modified Eagle’s medium (DMEM) was from PanEco (Moscow, Russia). Fetal bovine serum (FBS) was from HyClone (Logan, UT, USA). Endothelial cell growth medium (EGM) was from Cell Applications (#211-500, San Diego, CA, USA). Gelatin and Polybrene were from Sigma-Aldrich (St. Louis, MO, USA); glutamine, penicillin, and streptomycin were from Gibco (Waltham, MA, USA). MycoZap™ Plus-PR antibiotic formulation was from Lonza (Basel, Switzerland). Regular cell culture dishware was from SPL (Pocheon-si, South Korea); Ibidi (Gräfelfing, Germany) dishware was used for experiments where shear stress was applied.

Antibodies against the following proteins were used for Western blotting: vinculin (#ab18058) from Abcam (Cambridge, UK); glyceraldehyde phosphate dehydrogenase (GAPDH, #MAB374) from Merck (Rahway, NJ, USA); AMPK-α1 (#2795) and AMPK-α2 (#2757) from Cell Signaling (Danvers, MA, USA). HRP-conjugated goat antirabbit IgG (#7074, Cell Signaling, Danvers, MA, USA) and HRP-conjugated rabbit antimouse IgG (#A9044, Sigma-Aldrich, St. Louis, MO, USA) were used as secondary antibodies. Precision Plus prestained protein mixture and Clarity ECL reagent were from Bio-Rad (Hercules, CA, USA).

### 2.2. Cloning the Anti-Human AMPK-α shRNAs

Nucleotide sequences containing a 21 bp target were designed based on human *PRKAA1* (NM_006251) and *PRKAA2* (NM_006252) mRNA sequences. The selected target sequences for shRNAs were 5′-GAGTCTACAGTTATACCAAGT (AMPK-α1), 5′-GAAACGAGCAACTATCAAAG (AMPK-α2), and 5′-GATGTCAGATGGTGAATTT (AMPK-α1/2). The target and its reversed complementary sequences connected using a 4–6 base pair loop were subcloned into the pSIH3–CPHB vector at the unique BstV2I site. The loop sequences were 5′-CGAA for both AMPK-α1 and AMPK-α2 shRNAs or 5′-CAAGAG for the AMPK-α1/2 shRNA. All final constructs were verified using direct sequencing.

### 2.3. Cell Culture and Treatment

Human umbilical vein endothelial cells (HUVECs) were obtained from 3 healthy donors and mixed in equal proportions; passage 3 HUVECs were used in all experiments. HUVECs were grown to confluency in 60 mm dishes precoated with 0.2% gelatin in EGM containing 2 mM glutamine, 100 U/mL penicillin, and 100 μg/mL streptomycin. Human adipose microvascular endothelial cells (HAMECs) were isolated from visceral abdominal fat biopsies obtained from 3 morbidly obese females with T2D during bariatric surgery. Donor age (years)/BMI values were 33/47, 42/43, and 59/44. After collagenase digestion of fat tissue, endothelial cells were recovered using immunomagnetic selection on CD31 Dynabeads (Invitrogen, Carlsbad, CA, USA) and seeded on gelatin-coated petri dishes separately for each donor in EGM supplemented with MycoZap™ Plus-PR. Cells were propagated for 3–4 passages and combined in equal proportions to make the HAMEC pool used in this study. HEK293T cells were maintained in DMEM supplemented with 10% FBS, 2 mM glutamine, 100 U/mL penicillin, and 100 μg/mL streptomycin. The medium was replaced with a fresh one every other day. Cell images were acquired using an AxioVert 200M microscope equipped with a high-resolution AxioCam CCD camera (Zeiss, Oberkochen, Germany) and an on-stage incubator adjusted to 37 °C. Images were processed and analyzed using ImageJ 1.52a freeware (NIH, Bethesda, MD, USA).

BSA (20 mg/mL) or 8 mM PA/20 mg/mL BSA complex stock solutions were prepared in EGM and stored at 4 °C. Briefly, palmitate-Na was dissolved in 50% ethanol to a final concentration of 200 mM, heated to 60 °C, added to 20 mg/mL BSA in a 1 to 25 ratio (vol:vol), and mixed extensively. Ethanol was depleted to 0.02% final in a 30-kDa Amicon Ultra-15 filter using repeated concentration/EGM replenishment cycles.

### 2.4. Lentivirus Assembly and HUVEC Transduction

Lentiviral particles were assembled in HEK293T cells. A standard calcium-phosphate technique was used to cotransfect HEK293T cells with pMD2.G, pCMV-dR8.91 [50], and a pSIH3–CPHB-based plasmid coding was used for the shRNA of interest. The virus-containing medium was collected 36–48 h after transfection, cell debris was removed using centrifugation at 15,000× *g* for 10 min, and the supernatant fraction was used to infect HUVECs immediately or after storage at −70 °C for up to 1 month. Passage 3 HUVECs were grown to 70% confluency and incubated for 12–24 h with the virus-containing medium diluted with EGM (1:1) and supplemented with 8 μg/mL of Polybrene. Cells were used for experiments at least 5 days after the infection.

#### 2.4.1. Flow Cytometry

PA-induced cell surface exposure of proinflammatory markers ICAM-1 and phosphatidylserine, or intracellular activated caspase-3 (a marker of apoptosis), or propidium iodide staining (for analysis of cell membrane integrity) were measured using a FACSCanto II flow cytometer (Becton Dickinson, Franklin Lakes, NJ, USA) exactly as described previously [51].

#### 2.4.2. Cell Culture under Flow/Shear Stress

An ibidi Pump System equipped with a yellow-green perfusion set (Ibidi, Gräfelfing, Germany) was used to impose shear stress over confluent endothelial cells grown in gelatin-coated ibidi µ-slides I^0.4^ by generating unidirectional laminar flow of EGM supplemented with 1.1 mM palmitate (HUVEC) or 1.5 mM palmitate (HAMEC) through the µ-slide channel. Alternating 30 s cycles of laminar shear stress graded 5 dynes/cm^2^ or 10 dynes/cm^2^ were applied to cells to approximate the pulsatile pattern of blood flow in vasculature. Flow rates that generated the required shear stress were automatically calculated using the ibidi Pump Control v1.5.2 software for a given type of µ-slide and perfusion set used. Shear stress experiments were conducted in a CO_2_ incubator at 37 °C and 5% CO_2_ for time periods specified in the Results Section with periodical replenishing of sterile water in reservoirs with growth medium due to evaporation.

#### 2.4.3. Western Blotting

HUVECs were grown to confluency in 60 mm dishes precoated with 0.2% gelatin in EGM containing 2 mM glutamine, 100 U/mL penicillin, and 100 μg/mL streptomycin. The medium was decanted and the cells were washed briefly with ice-cold phosphate-buffered saline and lysed in 2× Laemmli sample buffer. The lysates were passed 20 times through a 30-gauge syringe needle to disrupt DNA, heated to 90 °C for 5 min, and clarified with centrifugation at 16,000× *g* at 4 °C for 10 min. The lysates were separated in 7.5% PAAG followed by a transfer to polyvinylidene fluoride filters. The filters were blocked in 5% nonfat milk and probed with antibodies against proteins of interest, and then with corresponding secondary HRP-conjugated antibodies. For AMPK-α isoforms, the same filter was reprobed after stripping off the antibodies from the previous visualization step. Clarity ECL reagent (Bio-Rad, Hercules, CA, USA) and a Fusion-SL 3500 WL instrument (Vilber Lourmat, Collégien, France) were used for protein band visualization. The images were processed and analyzed with ImageJ 1.52a freeware (NIH, Bethesda, MD, USA); protein content in the samples was normalized to the content of vinculin and/or GAPDH.

#### 2.4.4. Statistics

The data were analyzed using a two-tailed Mann–Whitney U-test with a significance level of 0.05. For multiple comparisons, a Kruskal–Wallis test was applied followed by the post hoc Dunn’s test using a Bonferroni correction. The data were presented as box and whisker plots or mean ± SD as indicated in the figure legends. All experiments were carried out at least in duplicates and repeated *n* times (indicated in the figure legends).

## 3. Results

### 3.1. AMPK-α1 Knockdown in HUVEC Leads to AMPK-α2 Accumulation

**Figure 1:**
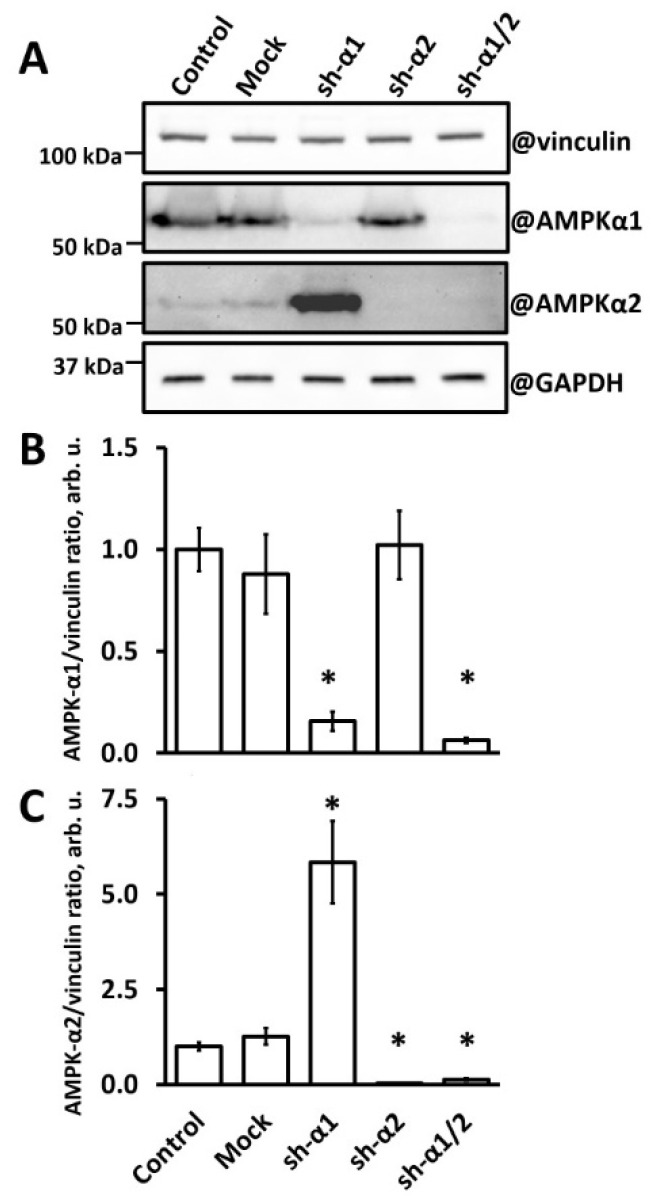
Knockdown of AMPK-α isoforms in HUVECs. AMPK-α1 knockdown leads to AMPK-α2 accumulation. Isoform-specific shRNA-mediated AMPK-α knockdown was achieved as described in the Methods Section; the content of AMPK-α isoforms was analyzed within 5 days after HUVEC transduction with lentiviruses coding for either AMPK-α1 (sh-α1), AMPK-α2 (sh-α2), or pan-AMPK-α1/2 (sh-α1/2) shRNA, or empty virus (Mock) and virus-naïve cells (Control). Representative Western blots (**A**) and statistics (**B**,**C**) drawn from two independent experiments after probing for AMPK-α1 (**B**) or AMPK-α2 (**C**) are shown. Vinculin and glyceraldehyde phosphate dehydrogenase (GAPDH) were used for protein loading controls as indicated; protein mass markers are indicated in the left. *, *p* < 0.01 vs. Control.

Our study design implied long-term monitoring of cells under palmitate-mediated lipotoxic stress with or without the simultaneous application of shear stress. Therefore, we chose the lentiviral shRNA-mediated modulation of the AMPK-α isoform levels since lentivirus integration in the genome and constitutive activity of the U6 promoter would allow for permanent knockdown in contrast to the temporary nature of siRNA-based gene silencing approaches [52]. Figure 1 shows the effective knockdown (KD) of either or both AMPK-α isoforms with the use of the corresponding specific shRNA. In AMPK-α1-KD HUVECs, the content of α1 decreased to 15% of that in control or mock-transduced cells. Meanwhile, these cells reciprocally enhanced the content of AMPK-α2 severalfold. As estimated by the pan-AMPK-α antibody, the level of total AMPK-α in AMPK-α1-KD cells was approximately 70% of that in mock or control cells, mostly representing AMPK-α2 immunoreactivity. In contrast, AMPK-α2-KD HUVECs showed no significant alteration in AMPK-α1 content (Figure 1). shRNA directed against both AMPK-α isoforms produced subtotal downregulation of AMPK-α1 and AMPK-α2 in HUVECs (Figure 1).

Thus, we produced HUVEC variants that contained predominantly either AMPK-α1 or AMPK-α2 in comparable amounts as well as HUVECs with substantially reduced levels of all catalytic subunits of the AMPK. Notably, cells with increased levels of AMPK-α2 acquired an elongated shape and became similar to HUVECs treated with AMPK activator 5-aminoimidazole-4-carboxamide ribonucleotide (AICAR) [41]. Cells with downregulated AMPK-α2 appeared larger than mock or control cells. Nevertheless, cells with either or both AMPK-α knockdowns were viable and formed monolayers, thus allowing for elucidation of the AMPK-α isoform’s involvement in the protection of endothelial cells from palmitate-induced lipotoxicity.

### 3.2. AMPK-α1 Is Critical for HUVEC Protection from the Palmitate Lipotoxicity

**Figure 2:**
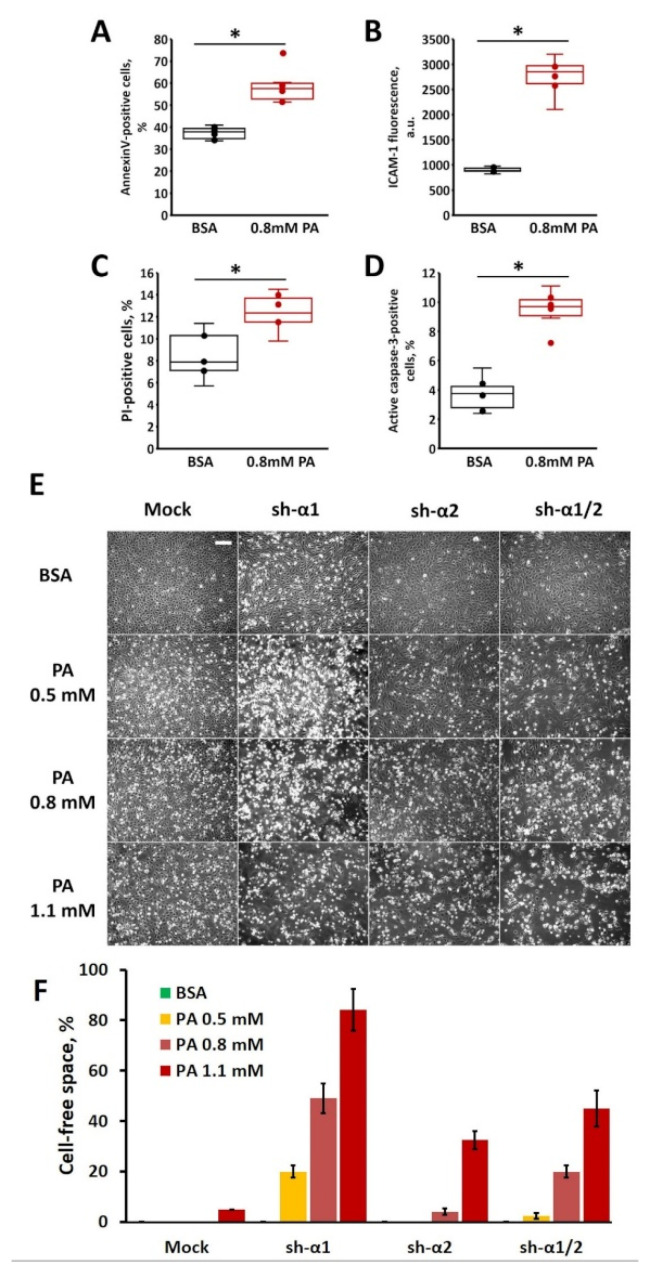
AMPK protects HUVECs from PA-induced lipotoxicity. Control HUVECs (**A**–**D**) or HUVECs with an isoform-specific knockdown (**E**,**F**) of AMPK-α1 (sh-α1), AMPK-α2 (sh-α2), both AMPK-α1/2 (sh-α1/2) or infected with an empty virus (Mock) as described in the Methods Section, were treated using the indicated concentrations of palmitic acid (PA) –BSA complex or control (BSA) for 2 days. Virus-naïve HUVECs were analyzed using flow cytofluorimetry (**A**–**D**) for phosphatidylserine (Annexin V-positive cells) (**A**) or ICAM-1 (**B**) exposure on the plasma membrane outer surface, propidium iodide (PI) staining (**C**), or active caspase-3 (**D**) as described in the Methods Section. The data are presented as box and whisker plots showing the interquartile range (**A**–**D**) for the percentage of cells positive for the corresponding marker (**A**,**C**,**D**) or fluorescence signal intensity (**B**). *, *p* < 0.01, *n* = 5–6. In AMPK-α-modulated HUVECs, monolayer integrity was estimated using phase-contrast microscopy at 10× magnification (**E**) and cell-free area calculation (**F**). The scale bar 200 µm applies to all images.

First, we tested the lipotoxic effects of the pathophysiologic PA concentration (0.8 mM). To directly assess the type of cell death caused by PA, cells were treated with 0.8 mM PA and evaluated via a classic annexin-V/propidium iodide (PI) protocol combined with staining of either ICAM-I as an inflammation-specific reporter, or active caspase-3 as an apoptosis-specific marker. As expected, 0.8 mM PA induced statistically significant increases in all parameters measured, i.e., a 1.5-fold increase was observed in the Annexin V-positive cell count (Figure 2A) indicating phosphatidylserine exposure to the outer cell surface as a marker of general cell activation, a 3-fold increase in ICAM-1 exposure on the cell surface (Figure 2B), a 1.5-fold increase in the count of cells permeant for PI (Figure 2C), and a 2.5-fold increase in activated caspase-3 (Figure 2D).

Next, we assessed the AMPK-α isoform-specific effects of PA in HUVECs under static conditions (Figure 2E). As expected, the cells displayed a dose-dependent response to PA. The knockdown of AMPK-α1 increased HUVEC sensitivity to PA, so after a 48 h incubation with as low as 0.5 mM PA, both AMPK-α1-KD and AMPK-α1/2-KD cells clearly displayed disturbed/disintegrated monolayers, while both mock or AMPK-α2-KD cells maintained apparently packed monolayers. Interestingly, cells that lacked only AMPK-α1 with a reciprocal increase in the AMPK-α2 level seemed to be even more sensitive to PA than cells lacking both α1 and α2 isoforms. In AMPK-α2-KD HUVECs, at 48 h, lipotoxic effects of PA were apparent at the highest PA concentration tested (1.1 mM) while the mock-infected cells were less severely affected. Altogether, these results indicate that AMPK-α1 is critical for HUVECs to sustain PA lipotoxicity and suggest that upregulation of AMPK-α2 may augment the lipotoxic response, although this possibility needs further examination.

### 3.3. Shear Stress Protects HUVEC from Palmitate Lipotoxicity Independently of AMPK

**Figure 3:**
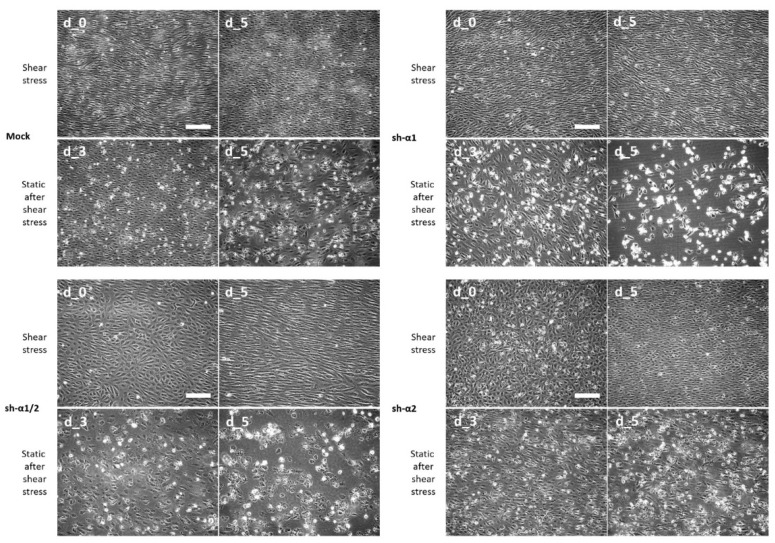
Shear stress protects HUVECs against palmitate lipotoxicity independently of AMPK. The cells expressing both α1 and α2 (mock), or none (sh-α1/2), or predominantly α2 (sh-α1), or predominantly α1 (sh-α2) AMPK isoforms were subjected for 2 days to laminar pulsatile flow (d_0 in each panel). Then, the medium was supplemented with 1.1 mM PA and the pulsatile flow continued for another 5 days (d_5, shear stress in each panel). Then, the cells were transferred to static conditions for another 5 days and imaged at day 3 (d_3) and day 5 (d_5, static after shear stress in each panel). Representative phase-contrast images were acquired at 10× magnification. The scale bar was 200 µm.

Keeping in mind that AMPK activity is upregulated by shear stress [32,33] and based on different contributions of AMPK-α isoforms to HUVEC resistance to lipotoxicity (Figure 2E), we explored the role of AMPK-α isoforms in shear stress-mediated protection. HUVECs were grown into monolayers and subjected to laminar shear stress of alternating amplitudes (5 and 10 dynes/cm^2^, 30 s each) for 2 days (Figure 3, shear stress, d_0). Under the shear stress conditions, all cells ultimately adopted elongated morphology and aligned in the direction of flow, although AMPK-α2 deficiency delayed cell alignment (Figure 3, shear stress, d_5).

Anticipating a strong protective effect of shear stress [8,21,23], we chose the highest PA concentration of the above tested under static conditions (i.e., 1.1 mM). Indeed, shear stress conferred strong protection against PA-mediated lipotoxicity, regardless of AMPK-α presence in HUVECs (Figure 3, compare the shear stress panels). Under shear stress, all AMPKα-modulated cells maintained integral monolayers oriented along the flow direction for at least 5 days, whereas simultaneously started static cultures died within 2–3 days at this PA concentration (Figure 2E).

Having undergone a 5-day-long shear stress in the presence of 1.1 mM PA, cells were transferred to static conditions with a fresh 1.1 mM PA supplementation. Under these conditions, the lipotoxic effect of PA re-emerged after a short lag (Figure 3, static after shear stress) resembling the pattern of cell viability deterioration seen under static conditions without intervening shear stress (c.f., Figure 2E). In HUVECs with AMPK-α1 deficiency (sh-α1 and sh-α1/2), the loss of monolayer integrity was conspicuous by day 3, whereas in mock controls and AMPK-α2-KD (sh-α2), the viability decline occurred slower (Figure 3, static after shear stress). At day 5 after shear stress cessation, the cell-free areas in AMPK-modulated monolayers were 73.1 ± 5.9% (sh-α1), 56.0 ± 1.4% (sh-α1/2), and 24.6 ± 1.2% (sh-α2). These findings indicate that the protective effect of shear stress is independent of AMPK, while basal AMPK activity reveals itself under static conditions, mainly via the AMPK-α1 isoform, which delays cell response to lipotoxicity. In addition, similar patterns of cell sustainability to PA before and after shear stress indicate that mechanical stimulation of endothelial cells did not affect shRNA interference reaction and downregulation of the AMPK.

### 3.4. Shear Stress Protects Diabetic Microvascular Endothelial Cells from Palmitate Lipotoxicity

**Figure 4:**
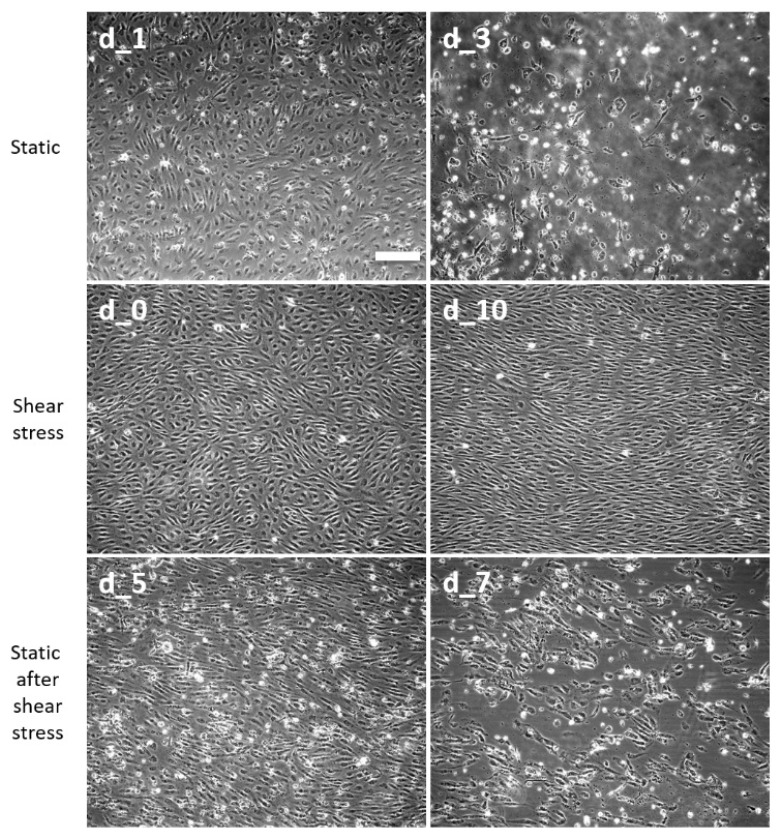
Shear stress protects diabetic human adipose microvascular endothelial cells (HAMECs) from PA-mediated lipotoxicity. HAMECs were obtained from three obese/T2D patients as described under the Methods Section, pooled, and either treated directly under static conditions with 1.5 mM PA for 1 (d_1) and 3 days (d_3, top, static), or subjected to laminar pulsatile shear stress of 5–10 dynes/cm^2^ for 2 days in the absence of PA (d_0), followed by 10 days in the presence of 1.5 mM PA (d_10, middle, shear stress), and then returned to static conditions for another 5 (d_5) or 7 (d_7) days (bottom, static after shear stress) with continued 1.5 mM PA treatment. Representative phase-contrast images were acquired at 10× magnification. The scale bar was 200 µm.

Obesity is associated with impaired endothelial function, low capillary density, and reduced blood flow in adipose tissue, with more pronounced defects attributed to the visceral fat compartment [3,4]. It was therefore plausible to explore the sensitivity of human adipose microvascular endothelial cells (HAMEC) to PA under static and flow conditions. HAMECs well tolerated 1.1 mM PA under shear stress as did HUVECs (Figure 3). Therefore, we increased the PA concentration to 1.5 mM PA. In static culture in the presence of 1.5 mM PA, subtotal cell death occurred by day 3 (Figure 4, top panels, cell-free area of approx. 80%), whereas cells were virtually unaffected by PA under shear stress for as long as 10 days (Figure 4, middle panels). When transferred back to static conditions in the presence of 1.5 mM PA, cell monolayers started to deteriorate by day 5 and to rarefy by day 7 (Figure 4, bottom panels, cell-free area of approx. 50%), demonstrating similar behavior to mock HUVECs.

## 4. Discussion

A number of reports have highlighted the possibility of HUVEC salvage in lipotoxic milieu through forced activation of the endothelial AMPK using low molecular weight substances including AICAR and forskolin or using the protein adiponectin [12,13,40,41]. Additionally, blood flow-generated shear stress is a widely recognized physiologic factor that stimulates AMPK activity in the endothelium [32,33,35] and exerts a strong protective antilipotoxic effect [27,31]. Oscillatory shear stress also upregulates AMPK in the endothelium of large vessels; however, whether this is a protective response to lipotoxicity is unknown [48]. Since two catalytic isoforms of AMPK, α1 and α2, are present in endothelial cells and are functionally nonredundant [42,44,53], it was our goal to elucidate which of these isoforms is involved in protection from lipotoxicity.

Using the shRNA interference, we achieved a profound and stable knockdown of the AMPK-α1 subunit in HUVECs. The AMPK-α1 knockdown was accompanied by a severalfold gain in AMPK-α2 content (Figure 1) suggesting that AMPK-α1 controls and limits AMPK-α2 expression. A similar relationship between AMPK-α1 and AMPK-α2 was reported in murine embryonic fibroblasts [54]. In contrast to our findings, no increase in AMPK-α2 was associated with AMPK-α1 deficiency after the siRNA-mediated knockdown of AMPK-α1 in HUVECs [55,56] or in the lung tissue of mice with an endothelium-specific AMPK-α1 knockout [45,57]. This discrepancy could have arisen from the different efficiencies of AMPK-α1 depletion and/or the timing of cell analysis because of the transitory nature of the siRNA-mediated knockdown; otherwise, it may reflect differences between macro and microvascular endothelial cells [58] or systemic compensation gained by the knockout animals. It should also be noted that many studies that applied the siRNA-mediated AMPK-α1 knockdown used a pan-AMPK-α antibody to analyze the knockdown effect, so the possible gains in the AMPK-α2 content could have occurred unrecognized [20,33,44,46]. Noteworthily, there was no reciprocal increase in the AMPK-α1 isoform following the knockdown of AMPK-α2 in HUVECs.

Endothelial cells that retained endogenous levels of AMPK-α1 (sh-α2 panels in Figure 2) demonstrated better survival in 0.5–0.8 mM PA than cells with an AMPK-α1 knockdown or an AMPK-α1/2 double knockdown. The AMPK-α2 knockdown had no effect on cell viability at these palmitate concentrations, consistent with the minor representation of the AMPK-α2 isoform in the endothelium [44]. In contrast, the increase in AMPK-α2 above endogenous levels that approximated AMPK-α1 levels in HUVECs failed to reproduce the protection provided by AMPK-α1 but resulted in the hypersensitivity of cells to the palmitate insult. Endothelial cells with the altered α1/α2 ratio toward α2 prevalence were prone to demise even at 0.5 mM palmitate, which is generally considered a physiologic palmitate level. Overall, the α1 catalytic subunit of AMPK but not the α2 isoform confers protection to HUVECs against palmitate lipotoxicity. However, this protection is limited and ineffective at higher palmitate concentrations unless AMPK is additionally stimulated [13,41].

Shear stress stimulates AMPK activity in the endothelium [32,33,35] and protects the endothelium from FFA lipotoxicity [27,31]. It is thus logical to assume that the AMPK mediates the antilipotoxic effect of shear stress and to address the question as to which AMPK-α isoform is involved in endothelium protection from lipotoxicity. In order to investigate this, we subjected HUVECs depleted in either α1 or α2, or both AMPK-α isoforms, to laminar pulsatile shear stress in vitro. Our experimental data suggest that although laminar shear stress is indeed a strong protector from lipotoxicity, depletion of either α1 or α2, or even both these isoforms, had no significant impact on the viability of HUVECs in the presence of high (1.1 mM) PA concentrations. Under shear stimulation, endothelial cells maintained a dense oriented monolayer for 5 days and longer, whereas in static but otherwise similar culture conditions cell monolayer integrity was already lost at day 2 due to progressive cell death. These data suggest that laminar shear stress-mediated protection of endothelial cells from lipotoxicity is not critically dependent on AMPK activation for relaying external mechanical stimuli into intracellular pathways and effector mechanisms that counteract lipotoxicity. Apart from AMPK activation, the mechanisms of antilipotoxic effects of shear stress may involve stearoyl-CoA desaturase-1 activation [31], mitochondrial biogenesis [27], shear stress-mediated regulation of death-associated protein kinase [24], alteration in EC metabolism [25], and multiple reprogramming events on the gene expression level [23]. For future studies, it may be of interest to explore whether turbulent/oscillatory vs. laminar shear stress is associated with lower local AMPK activity in the vascular endothelium.

Once the flow over endothelial cells ceased and mechanostimulation stopped, AMPK-α1-dependent transient protection from palmitate toxicity reappeared, recapitulating cell viability effects observed in static culture. Cells with a knockdown of AMPK-α1 demonstrated accelerated death compared with mock controls and cells with AMPK-α2 knockdown. Altogether, this suggests that AMPK-α1-controlled antilipotoxic machinery coexists in endothelial cells with other shear stress-dependent protective mechanisms and requires additional AMPK activation for efficient work.

The laminar shear stress-dependent salvage from pathologic levels of palmitate was successfully reproduced in microvascular endothelial cells from visceral adipose tissue (HAMECs) of subjects with morbid obesity and T2D who experience metabolic stress and elevated FFA levels in the blood, in particular. In static culture conditions before and after shear stress stimulation, HAMECs behaved similarly to HUVECs. Although HAMECs appeared more resistant to palmitate challenge, they also demonstrated progressive cell death when presented with appropriately high palmitate concentrations. In our experiments, 1.5 mM PA was detrimental to HAMEC viability in static cell culture but did not affect these cells in conditions of growth medium flow.

Obesity is associated with impaired endothelial function, low capillary density, and reduced blood flow in adipose tissue, with more pronounced defects attributed to the visceral fat compartment [3,4]. Our in vitro findings regarding visceral fat HAMECs in static and mechanically active environments suggest that capillaries with reduced or absent blood circulation in obese/T2D subjects may be prone to irreversible endothelial damage from elevated plasma FFA, with consequent desquamation from the basal membrane and activation of thrombotic events on exposed extracellular matrix proteins. Such microvascular alterations in combination with hyperglycemia-induced endothelial damage may account for vasculopathies typical for advanced T2D. Our experiments also indicate that the onset of endothelial cell demise after cessation of flow/shear stress is not immediate, suggesting that lipotoxic effects are unlikely to manifest after short interruptions in microvascular blood flow in vivo. However, prolonged deprivation from microcirculation-stimulating physical activity may be harmful to the microvessels of heavily obese and diabetic subjects.

**Figure 5:**
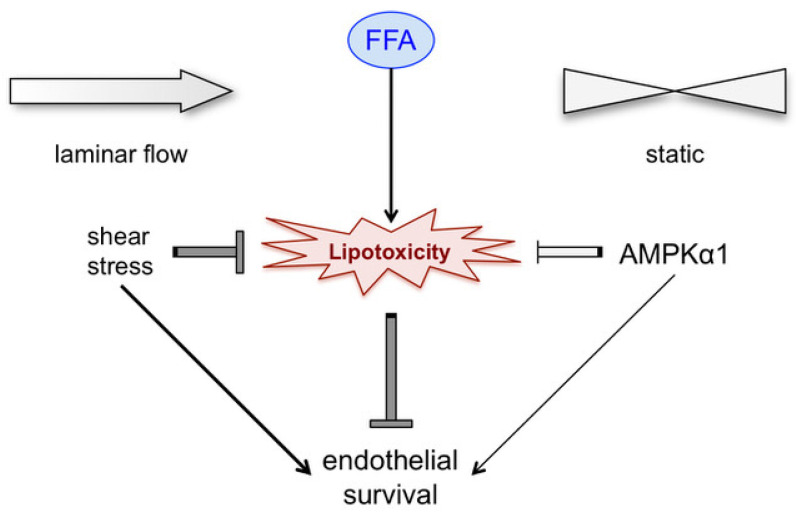
Proposed involvement of shear stress and the AMPK in protection of the vascular endothelium against lipotoxicity emerging from the results of this study. While shear stress protects endothelial cells under flow conditions, the AMPK protects them, though less efficiently, under static conditions. This coordinated mechanism may confer protection to microvessels with restricted circulation in obesity/T2D. Its dysfunction due to AMPK impairment may contribute to diabetes-associated vascular complications.

In summary, we demonstrated that AMPK and shear stress independently protect endothelial cells from palmitate lipotoxicity (Figure 5). Of two catalytic AMPK-α isoforms expressed in endothelial cells, AMPK-α1 is involved in protection whereas AMPK-α2 does not substitute AMPK-α1 in its protective role. Microvascular endothelial cells from obese and diabetic subjects are prone to high palmitate-induced damage and demise in static cell culture but acquire long-term protection from excessive palmitate toxicity when subjected to laminar flow/shear stress. Maintaining active microcirculation through physical activity may be a viable approach to attenuate microvascular damage in obese/T2D subjects.

## Data Availability

The datasets created during the current study are available from the corresponding authors upon reasonable request.
